# Applicability of scoring systems predicting outcome of transarterial chemoembolization for hepatocellular carcinoma

**DOI:** 10.1007/s00432-020-03135-8

**Published:** 2020-02-27

**Authors:** Marie Vogeler, Isabelle Mohr, Jan Pfeiffenberger, Simon David Sprengel, Miriam Klauss, Andreas Teufel, De-Hua Chang, Christoph Springfeld, Thomas Longerich, Uta Merle, Arianeb Mehrabi, Karl Heinz Weiss, Markus Mieth

**Affiliations:** 1 Internal Medicine IV, Heidelberg University Hospital, Heidelberg, Germany; 2 Department of Radiology, Heidelberg University Hospital, Heidelberg, Germany; 3 Department of Medical Oncology, Heidelberg University Hospital, National Center for Tumor Diseases (NCT), Heidelberg, Germany; 4 Department of Pathology, Heidelberg University Hospital, Heidelberg, Germany; 5 Department of General, Visceral and Transplantation Surgery, Heidelberg University Hospital, INF 110, 69120 Heidelberg, Germany; 6 Division of Hepatology, Department of Medicine II, Medical Faculty Mannheim, Heidelberg University, Mannheim, Germany; 7 Liver Cancer Center Heidelberg (LCCH), Heidelberg University Hospital, Heidelberg, Germany

**Keywords:** TACE, Embolization, Liver cancer, Stage migration

## Abstract

**Purpose:**

Several scoring systems have been proposed to predict the outcome of transarterial chemoembolization (TACE) in patients with hepatocellular carcinoma (HCC). However, the application of these scores to a bridging to transplant setting is poorly validated. Evaluation of the applicability of prognostic scores for patients undergoing TACE in palliative intention vs. bridging therapy to liver transplantation (LT) is necessary.

**Methods:**

Between 2008 and 2017, 148 patients with HCC received 492 completed TACE procedures (158 for bridging to transplant; 334 TACE procedures in palliative treatment intention at our center and were analyzed retrospectively. Scores (ART, CLIP, ALBI, APRI, SNACOR, HAP, STATE score, Child–Pugh, MELD, Okuda and BCLC) were calculated and evaluated for prediction of overall survival. ROC analysis was performed to assess prediction of 3-year survival and treatment discontinuation.

**Results:**

In patients receiving TACE in palliative intention most scores predicted OS in univariate analysis but only mSNACOR score (*p* = 0.006), State score (*p* < 0.001) and Child–Pugh score (*p* < 0.001) revealed statistical significance in the multivariate analysis. In the bridging to LT cohort only the BCLC score revealed statistical significance (*p* = 0.002).

**Conclusions:**

Clinical usability of suggested scoring systems for TACE might be limited depending on the individual patient cohorts and the indication. Especially in patients receiving TACE as bridging to LT none of the scores showed sufficiently applicability. In our study Child–Pugh score, STATE score and mSNACOR score showed the best performance assessing OS in patients with TACE as palliative therapy.

## Introduction

Therapeutic approaches to hepatocellular carcinoma (HCC) are multimodal. Management and prognosis of HCC patients highly depends on tumor status, general health and actual liver functional reserve (Cabibbo et al. 2010; Llovet et al. 1999b; Marrero et al. 2005; Okuda et al. 1985). Curative treatments in terms of resection, liver transplantation or local ablation are often restricted to subgroups with preserved liver function and limited tumor load (Bruix and Sherman 2005; Llovet et al. 2005; 2012). For intermediate stage HCC patients, TACE is currently considered (palliative) first line-therapy (Bruix and Sherman 2011; Llovet and Bruix 2003; Llovet et al. 2008) offering local tumor control and prolongation of OS (Arii et al. 2000; Ikai et al. 2004; Lee et al. 2012; Takayasu et al. 2006). Apart from its use in intermediate and advanced tumor stages, another application for TACE is as bridging treatment to liver transplantation (Decaens et al. 2005; Llovet et al. 2012; Kollmann et al. 2017; Majno et al. 1997; Porrett et al. 2006; Bruix et al. 2011). Various scoring systems (Table 1) predicting the prognosis of HCC patients undergoing different therapies are available (Ho et al. 2017; Hucke et al. 2014a, b; Kadalayil et al. 2013; Kamath et al. 2001; Kim et al. 2016; Li et al. 2016; Marrero et al. 2005; Cancer of the Liver Italian Program (CLIP) Investigators 1998; Okuda et al. 1985; Sawhney et al. 2011; Song et al. 2016; Yin et al. 2016), to guide treatment decisions, like e.g., the commonly used BCLC classification (Cillo et al. 2006; Guglielmi et al. 2008; Llovet et al. 1999a, 2008,2012; Marrero et al. 2005; Vitale et al. 2009). In the setting of TACE a considerable number of scores, such as Child–Pugh (Child and Turcotte 1964; Pugh et al. 1973), ALBI (Johnson et al. 2015), APRI (Song et al. 2016; Wai et al. 2003), HAP (Kadalayil et al. 2013), ART (Sieghart et al. 2013), CLIP (Cancer of the Liver Italian Program Investigators 1998), SNACOR (Kim et al. 2016), MELD (Kamath et al. 2001; Sawhney et al. 2011), Okuda (Okuda et al. 1985) and STATE (Hucke et al. 2014a) aim to predict prognosis of HCC patients undergoing therapy. But especially data on a bridging to transplant collective or comparative data between scores are sparse. The current study retrospectively assessed the proposed scoring systems in HCC patients eligible for TACE for bridging to transplant or in palliative.

**Table 1:** Assessed scores in this study

Score	Included variables	Prognostic groups identified by the score	Median OS (months)^b^	References
BCLC (Biolato 2014)	ECOG score	A (early stage)	18.3–76.2	Abbasi et al. (2017), Allgaier et al. (1998) and Arii (2000)
	Number and diameter	B (intermediate stage)	15.1–24	Abbasi et al. (2017), Allgaier et al. (1998) and Arii (2000)
	Vascular invasion and metastasis	C (advanced stage)	9–13.7	Abbasi et al. (2017), Allgaier et al. (1998) and Arii (2000)
	Child–Pugh score	D (terminal stage)	4–5.4	Abbasi et al. (2017), Allgaier et al. (1998) and Arii (2000)
	Okuda score			Abbasi et al. (2017), and Arii (2000)
Child–Pugh (Child and Turcotte 1964, Cholongitas et al. 2005)	Serum albumin	A = 5–6 points	18.3–104	Abbasi et al. (2017), Arii (2000), Bruix and Sherman (2005), Bruix et al. (2011), Bruns et al. (2014) and Cabibbo et al. (2010)
	INR	B = 7–9 points	11.8–46	Arii (2000), Bruix and Sherman (2005), Bruix et al. (2011), Bruns et al. (2014) and Cabibbo et al. (2010)
	Ascites	C ≥ 10 points	4–23.8	Arii (2000), Bruix and Sherman (2005), Bruns et al. (2014) and Cabibbo et al. (2010)
	Encephalopathy			
	Total bilirubin			
ART (Abbasi et al. 2017)	Child–Pugh score	Low risk < 2.5 points	23.1–104	Abbasi et al. (2017), Bruix et al. (2011), Cillo (2004,2006)
	Radiologic tumor response	High risk ≥ 2.5 points	5.4–25	Abbasi et al. (2017), Bruix et al. (2011), Cillo (2004,2006)
	AST			
HAP (Dhanasekaran et al. 2010)	AFP	A = 0 points	25.5—n.r.	Bruix et al. (2011), Cabibbo et al. (2010), Cillo (2004), Dhanasekaran et al. (2010), Durand and Valla (2008) and Llovet et al. (2012)
	Serum albumin	B = 1 point	18.1–55.0	Bruix et al. (2011), Cabibbo et al. (2010), Cillo (2004), Dhanasekaran et al. (2010), Durand and Valla (2008) and Llovet et al. (2012)
	Total bilirubin	C = 2 points	8.9–46.0	Bruix et al. (2011), Cabibbo et al. (2010), Cillo (2004), Dhanasekaran et al. (2010), Durand and Valla (2008) and Llovet et al. (2012)
	Diameter	D ≥ points	3.6–18	Bruix et al. (2011), Cabibbo et al. (2010), Cillo (2004), Dhanasekaran et al. (2010), Durand and Valla (2008) and Llovet et al. (2012)
STATE (El Khaddari et al. 2002)	CRP	Low risk ≥ 18 points	19.5–22.2	El Khaddari et al. (2002), Farinati et al. (2000) and Georgiades et al. (2006)
	Up-to-seven criteria	High risk < 18 points	5.3–14.3	El Khaddari et al. (2002), Farinati et al. (2000) and Georgiades et al. (2006)
	Serum albumin			
APRI (Guglielmi et al. 2008, Hinrichs 2017)	Platelet count	≤ 1.15^a^	(–)	
	AST	> 1.15^a^	(–)	
ALBI	Total bilirubin	A1 ≤ − 2.6	28.9–38.9	Cabibbo et al. (2010), Ho (2017)
	Serum albumin	A2 > − 2.6 ≤ − 1.39	10.2–22.4	Cabibbo et al. (2010), Ho (2017) and Hucke (2014a)
		A3 > − 1.39	6.05–15.3	Cabibbo et al. (2010) and Hucke (2014a)
SNACOR (Hucke et al. 2014b)	Number and Diameter	Low risk 0–2 points	31.5–49.8	Hucke et al. (2014b) and Ikai (2004)
	AFP	Interm. risk 3–6 points	19.9–30.7	Hucke et al. (2014b) and Ikai (2004)
	Child–Pugh class	High risk 7–10 points	9.2–12.4	Hucke et al. (2014b) and Ikai (2004)
	Radiologic tumor response			
CLIP (Kadalayil 2013)	AFP	0 = 0 points	31–68.7	Arii (2000), Bruix and Sherman (2005), Bruns et al. (2014) and Johnson (2015)
	Portal vein thrombosis	1 = 1 point	27–43.8	Arii (2000), Bruix and Sherman (2005), Bruns et al. (2014) and Johnson (2015)
	Child–Pugh class	2 = 2 points	13–26.4	Arii (2000), Bruix and Sherman (2005), Bruns et al. (2014) and Johnson (2015)
	Number and diameter	3 = 3 points	8–15.0	Arii (2000), Bruix and Sherman (2005), Bruns et al. (2014) and Johnson (2015)
		4 ≥ 4 points	2–3.3	Arii (2000), Bruix and Sherman (2005), Bruns et al. (2014)
Okuda (Kamath 2001)	Tumor size	I = 0 points	27–45.5	Arii (2000), Bruix and Sherman (2005), Bruns et al. (2014) and Johnson (2015)
	Ascites	II = 1–2 points	10–21	Arii (2000), Bruix and Sherman (2005), Bruns et al. (2014) and Johnson (2015)
	Albumin	III = 3–4 points	2–16	Arii (2000), Bruix and Sherman (2005), Bruns et al. (2014) and Johnson (2015)
	Bilirubin			
MELD (Kim 2016; Kollmann et al. 2017)	INR	< 10	(–)	
	Creatinine	≥ 10	(–)	
	Bilirubin			

^a^Cut-off value concerning liver function deterioration after TACE

^b^Calculated in cohorts in which a minimum of 50% of the included patients were treated with TACE

(–)No information found about median OS with this cut-off value (in cohorts with a minimum of 50% TACE-treated patients)

## Materials and methods

### Study design

The retrospective cohort study was conducted in a tertiary care center (Heidelberg University Hospital) and was a priori approved by the institutional review board (IRB). Data collection was based on chart review. We included patients with established diagnosis of hepatocellular carcinoma according EASL criteria, who received at least one TACE as a therapy of HCC between 2011 and 2017 in our center (Llovet et al. 2012). Decision for TACE treatment and modality of beads (DEB-TACE, conventional TACE or TACE with biodegradable Particles) was in all cases assessed by a multidisciplinary tumor board. The boards treatment approach followed the current EASL-EORTC Clinical Practice Guidelines (Llovet et al. 2012) in patients who have unresectable lesions and are not suitable to receive other ablative therapies. Patients who had been diagnosed as BCLC stage A, C or D, but were unable or unwilling to receive the proposed therapy (e.g., LT, RF, Sorafenib) were also eligible for TACE therapy. For patients on the liver transplantation list TACE was considered standard bridging treatment.

### Subgroup definition

**Fig. 1:**
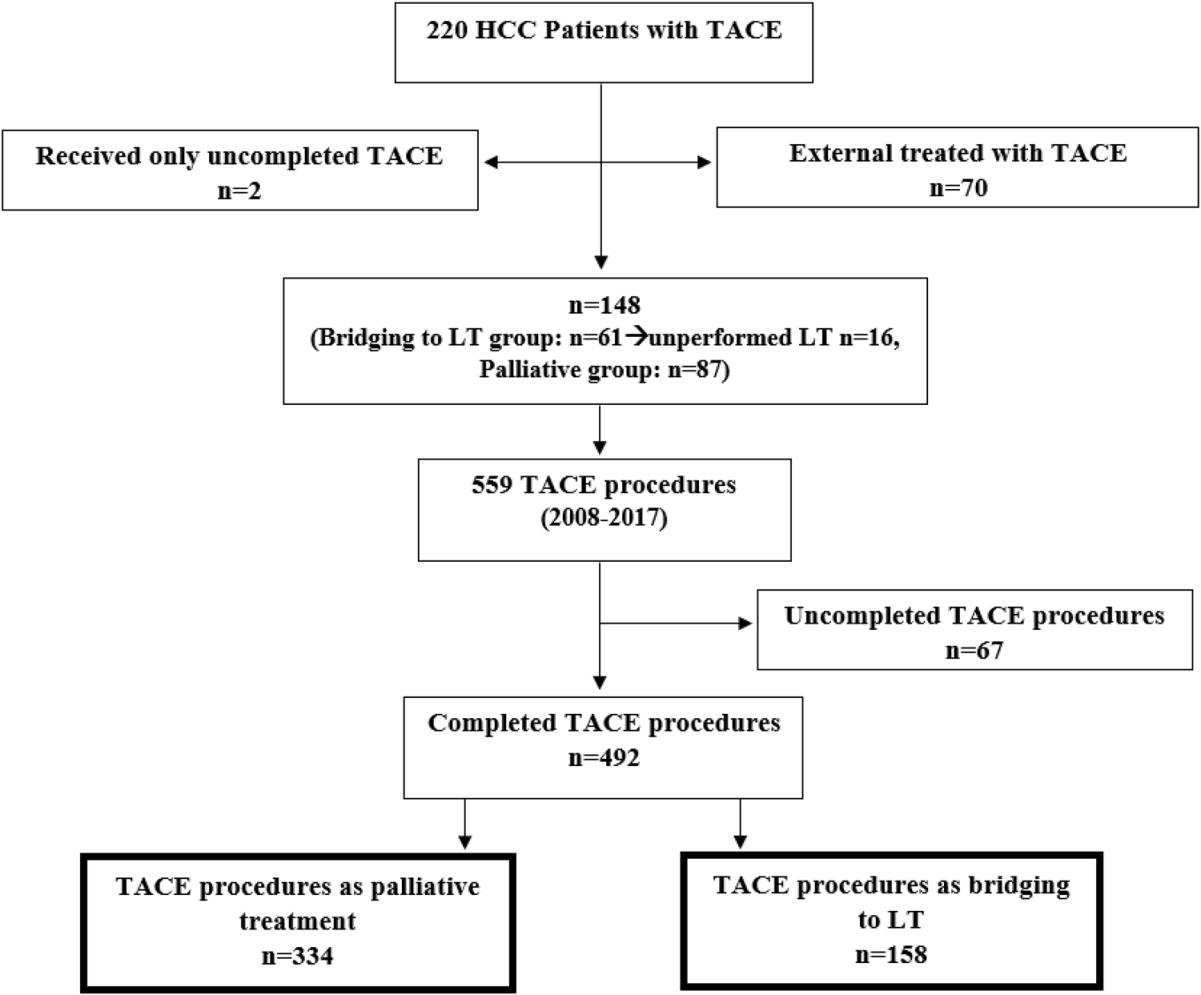
Study population

Each TACE procedure of the included patients was categorized in two different subgroups, depending on the treatment plan at the time of TACE therapy (Fig. 1): Bridging to transplant or palliative therapy. The bridging to LT dataset included all interventions in which patients were enrolled on the transplant waiting list at time of TACE, regardless of whether the LT was performed afterwards. The palliative dataset consisted of interventions performed in patients who did not meet the criteria for a liver transplant at the time of TACE.

### Calculation of scores

Scores were calculated at each TACE session. Score calculation was done according to their original formula. In addition, we calculated a modified SNACOR (mSNACOR) score and modified ART (mART) score. The original calculation of these two scores only includes parameters in comparison to the first TACE to assess whether a second TACE should be performed. To assess these scores with respect to each individual TACE, these parameters were calculated in comparison to the previous TACE instead to the first TACE.

### Statistics

Statistical analysis was performed using SPSS-25 software (IBM, Germany). The two-tailed Chi-squared was employed to compare categorical data of bridging dataset to palliative dataset. The Mann–Whitney *U* test was used for continuous variables. The primary endpoint was overall survival concerning the different scores, analyzed by Kaplan–Meier method and compared by log rank test. Significant scoring systems in the univariate analysis were introduced to multivariate Cox regression model to determine the adjusted risk ratio. The ROC analysis examines which score reflects best probability of achieving 3-year survival or probability of treatment discontinuation due to adverse events or death. 3-years survival was calculated from the time of each individual TACE. Statistical significance was set at *p* value < 0.05 in two-tailed tests.

## Results

### Patient characteristics and distribution of scores at TACE procedures

A total of 492 TACE sessions were included in this study (158 bridging/334 palliative sessions). In consequence of listing criteria, patients in the bridging cohort were younger, had a limited tumor disease and different tumor properties, such as less frequent portal or hepatic vein infiltration and no extrahepatic tumor manifestation. In the palliative dataset, 28 (8.4%) procedures were performed as conventional TACE with Carboplatin or Doxorubicin as chemotherapeutic agent combined with Lipiodol^®^, which is only half as common as in the bridging dataset. In the palliative group 73 (21.9%) TACE sessions were performed in patients who finally discontinued TACE therapy (and received no further local therapy) because of adverse events or death, whereas in the bridging dataset none of the patients discontinued the TACE therapy (Table 2). Median overall survival after TACE was not reached in the bridging dataset due to LT and was 21.8 months in the palliative dataset (Table 4). The descriptive comparative analysis of the scoring systems between both datasets is thus confounded by the different baseline characteristics and showed significant distinct distributions of the scoring values (BCLC stage, Child–Pugh class, STATE score, HAP stage, SNACOR stage, mSNACOR, ALBI group, CLIP group and MELD score) shown in Table 3. Only the APRI score, Okuda score, mART and the ART score did not differ significantly between the two subgroups (Table 3). Comparing the three most frequent etiologies (viral, alcoholic and cryptogenic/NASH in descending order) in ROC analysis with primary endpoint “3 years survival” and “treatment discontinuation” the scores revealed etiology as a potential confounding factor (Tables 8, 9).

**Table 2:** Patient characteristics at TACE procedures in different subgroups

Patient characteristics at TACE procedures	Bridging dataset		Palliative dataset		*p*
	*N* (%)	Total (*N*)	*N* (%)	Total (*N*)	
Number of all TACE procedures	158 (100)		334 (100)		
Age (years) (median, range)	57 (28–70)	158	71 (49–90)	334	< 0.001
Gender					
Male	127 (80.4)	158	249 (74.6)	334	0.155
Female	31 (19.6)		85 (25.4)		
Number of tumor nodules					
Single tumor nodule	61 (38.6)	158	64 (19.2)	334	< 0.001
> 1 tumor nodule	97 (61.4)		270 (80.8)		
Max. diameter, cm (median, range)	2.3 (0.9–5.0)	158	4.6 (0.6–19.3)	334	< 0.001
Liver vein infiltration	0 (0)	158	7 (2.1)	334	0.067
Portal vein infiltration	0 (0)	158	19 (5.7)	334	0.002
Vena cava infiltration	0 (0)	158	3 (0.9)	334	0.232
Extrahepatic tumor manifestation	0 (0)	158	40 (12.0)	334	< 0.001
Category of TACE treatment					
DEB	104 (65.8)	158	244 (73.1)	334	0.001
Conventional	32 (20.3)		28 (8.4)		
Biodegradable	22 (13.9)		62 (18.6)		
Additional therapy with sorafenib	19 (12.0)	158	16 (4.8)	334	0.004
Number of therapy discontinuations (not discontinuations of TACE procedures) after TACE due to AE or death	0 (0)	158	73 (21.9)	334	< 0.001

**Table 3:** Distribution of scores at TACE procedures in different subgroups

Distribution of scores at TACE procedures	Bridging dataset		Palliative dataset		*p*
	*N* (%)	Total (*N*)	*N* (%)	Total (*N*)	
BCLC stage					
A	103 (65.2)	158	52 (15.6)	334	< 0.001
B	29 (18.4)		182 (54.5)		
C	4 (2.5)		70 (21.0)		
D	22 (13.9)		30 (9.0)		
Child–Pugh class					
A	69 (48.9)	141	185 (64.9)	285	0.005
B	50 (35.5)		75 (26.3)		
C	22 (15.6)		25 (8.8)		
STATE score					
≥ 18	131 (92.9)	141	200 (70.9)	282	< 0.001
< 18	10 (7.1)		82 (29.1)		
HAP stage					
A	25 (18.0)	139	68 (25.2)	270	< 0.001
B	42 (30.2)		81 (30.0)		
C	71 (51.1)		89 (33.0)		
D	1 (0.7)		32 (11.9)		
mSNACOR stage					
Low risk	21 (23.9)	88	16 (8.2)	195	< 0.001
Interm. risk	67 (76.1)		146 (74.9)		
High risk	0 (0)		33 (16.9)		
SNACOR stage					
Low risk	11 (26.2)	42	10 (19.6)	51	0.026
Interm. risk	31 (73.8)		33 (64.7)		
High risk	0 (0)		8 (15.7)		
ALBI group					
A1	34 (24.1)	141	104 (36.7)	283	0.003
A2	84 (59.6)		157 (55.5)		
A3	23 (16.3)		22 (7.8)		
CLIP group					
0	19 (13.7)	139	32 (11.9)	270	< 0.001
1	63 (45.3)		72 (26.7)		
2	42 (30.2)		88 (32.6)		
3	14 (10.1)		47 (17.4)		
4	1 (0.7)		31 (11.5)		
APRI score					
< 1.15	58 (39.2)	148	140 (47.5)	295	0.099
> 1.15	90 (60.8)		155 (52.5)		
mART score					
≤ 1.5	42 (60.0)	70	93 (65.5)	142	0.434
> 2.5	28 (40.0)		49 (34.5)		
ART score					
≤ 1.5	22 (57.9)	38	29 (61.7)	47	0.722
≥ 2.5	16 (42.1)		18 (38.3)		
Okuda stage					
I	75 (53.2)	141	125 (43.9)	285	0.112
II	62 (44.0)		143 (50.2)		
III	4 (2.8)		17 (6.0)		
MELD score					
< 10	73 (49.3)	148	190 (64.0)	297	0.003
≥ 10	75 (50.7)		107 (36.0)		

### Performance of scores in the palliative dataset

#### Median overall survival (OS)

**Fig. 2:**
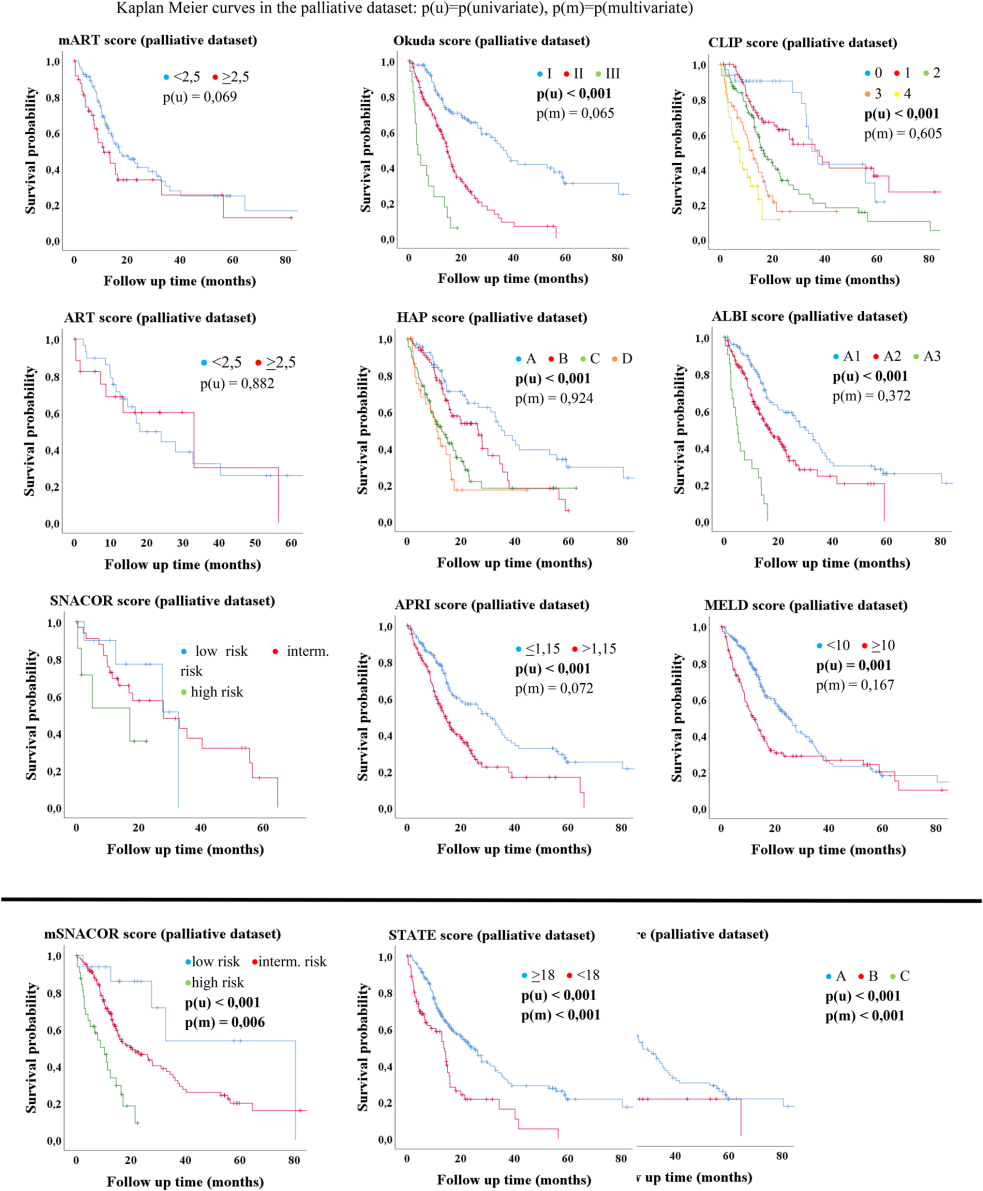
Kaplan–Meier-analysis: palliative dataset

**Fig. 3:**
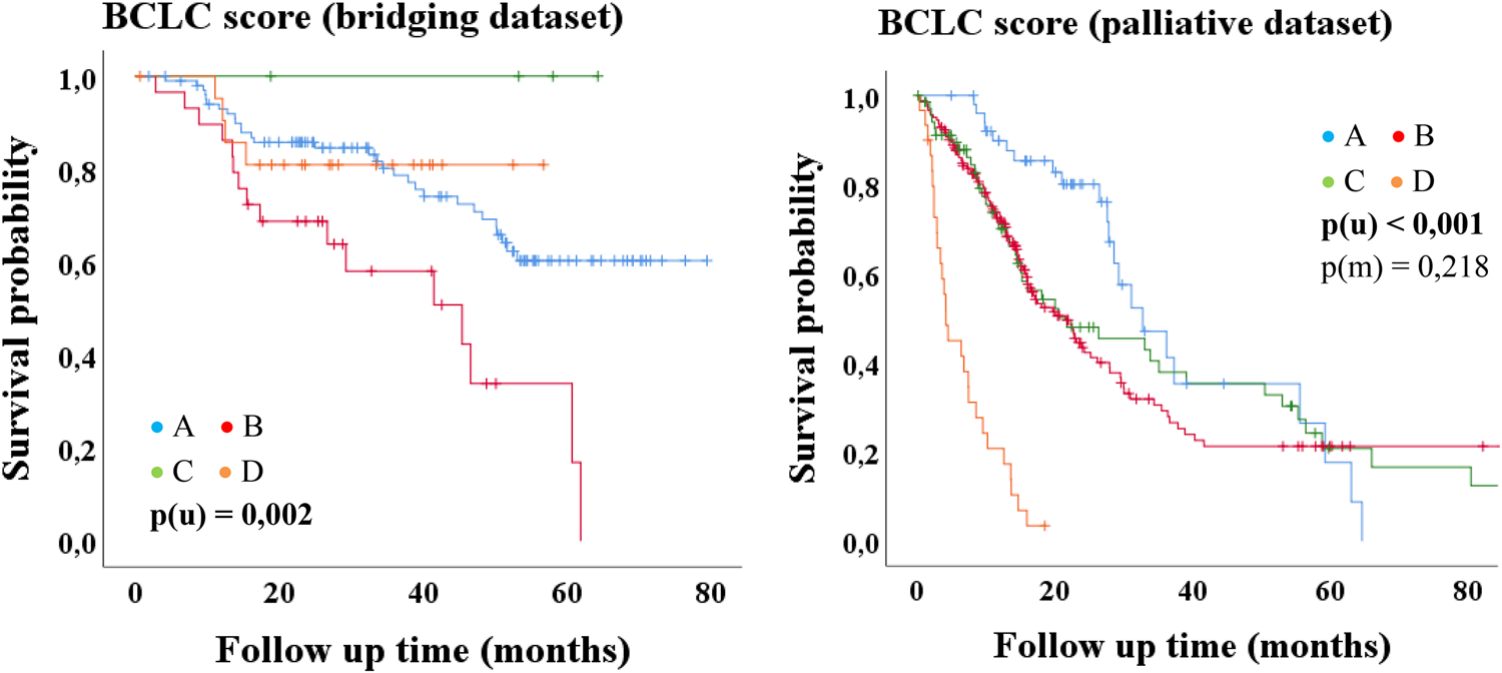
Kaplan–Meier-analysis: BCLC score

The univariate Kaplan–Meier analysis in the palliative dataset showed significant differences of median OS in majority of scores (Table 4, Figs. 2, 3). The ART, mART and SNACOR score were the only three scores that showed no significant results in univariate analysis. In multivariate analysis, only three scores were statistically significant independent parameters for the assessment of median OS. These were the Child–Pugh score, the STATE score and the mSNACOR score (which was calculated for each TACE treatment) (Table 4, Fig. 2).

**Table 4:** Uni- and multivariate analysis of scores in bridging vs. palliative dataset

Score	Bridging dataset Median OS, months (SD; 95% CI)	*p* (u/m)	Palliative dataset Median OS, months (SD; 95% CI)	*p* (u/m)
CLIP group				
0	Not reached	0.335/–	37.4 (3.1; 31.4–43.4)	< 0.001/0.605
1	Not reached		38.0 (7.4; 23.5–52.4)	
2	Not reached		16.7 (2.3; 12.1–21.3)	
3	Not reached		12.6 (1.9; 8.8–16.3)	
4	Not reached		7.5 (1.9; 3.7–11.3)	
mART score				
< 2.5	Not reached	0.472/–	17.2 (3.9; 9.6–24.8)	0.069/
≥ 2.5	Not reached		11.1 (2.6; 5.9–16.3)	
ART score				
< 2.5	Not reached	0.110/–	18.1 (5.0; 8.4–27.8)	0.882/
≥ 2.5	39.0^b^		33.1 (14.5; 4.7–61.6)	
ALBI group				
A1	Not reached	0.360/–	30.1 (3.4; 23.4–36.8)	< 0.001/0.372
A2	Not reached		16.9 (2.3; 12.3–21.5)	
A3	Not reached		5.1 (0.9; 3.3–6.8)	
APRI score				
≤ 1.15	Not reached	0.396/–	31.2 (3.5; 24.4–38.0) 14.5 (1.4; 11.8–17.2)	< 0.001/0.072
> 1.15	Not reached		31.2 (3.5; 24.4–38.0) 14.5 (1.4; 11.8–17.2)	
mSNACOR stage				
Low risk	51.8 (12.3; 27.6–75.9)	0.371/–	80.5^b^	< 0.001/0.006
Interm. risk	Not reached		20.6 (4.7; 11.5–29.8)	
High risk	No cases		10.4 (2.5; 5.4–15.3)	
SNACOR stage				
Low risk	39.0 (10.9; 17.6–60.4)	0.380/–	32.8^b^	0.253/
Interm. risk	60.8^b^		28.0 (9.9; 8.6–47.4)	
High risk	No cases		17.2 (8.6; 0.3–34.1)	
HAP stage				
A	Not reached	0.687/–	35.2 (3.6; 28.1–42.2)	< 0.001/0.924
B	Not reached		26.6 (4.8; 17.2–35.9)	
C	Not reached		13.0 (2.0; 8.9–17.0)	
D	Not reached		11.1 (1.8; 7.5–14.7)	
STATE score				
≥ 18	Not reached	0.804/–	24.1 (2.7; 18.8–29.3)	< 0.001/< 0.001
< 18	45.5 (23.8; 0–92.2)		13.7 (0.9; 12.1–15.4)	
Child–Pugh class				
A	Not reached	0.473/–	27.9 (3.2; 21.7–34.1)	< 0.001/< 0.001
B	Not reached		11.2 (1.5; 8.2–14.1)	
C	Not reached		4.2 (0.5; 3.2–5.3)	
BCLC stage				
A	Not reached	0.002/–	32.8 (4.4; 24.2–41.5)	< 0.001/0.218
B	45.5 (12.4; 21.1–69.8)		21.6 (2.5; 16.7–26.6)	
C	Not reached		21.8 (8.8; 4.6–39.1)	
D	Not reached		4.2 (0.5; 3.3–5.2)	
Okuda stage				
I	Not reached	0.330/–	36.3 (3.1; 30.2–42.4)	< 0.001/0.065
II	Not reached		14.7 (1.0; 12.7–16.6)	
III	Not reached		3.5 (1.1; 1.3–5.6)	
MELD				
< 10	Not reached	0.213/–	25.3 (2.5; 20.4–30.1)	0.001/0.167
≥ 10	62.0 (8.8; 44.8–79.1)		12.6 (1.7; 9.2–15.9)	
Total^a^	Not reached		21.8 (2.7; 16.6–27.1)	< 0.001

Kaplan–Meier and Cox Regression; *p*(u/m) = *p*(univariate analysis)/*p*(multivariate analysis); *SD* standard deviation, *95% CI* 95% confidence interval

^a^Excluded from multivariate analysis

^b^Not enough cases

#### Treatment discontinuation

**Fig. 4:**
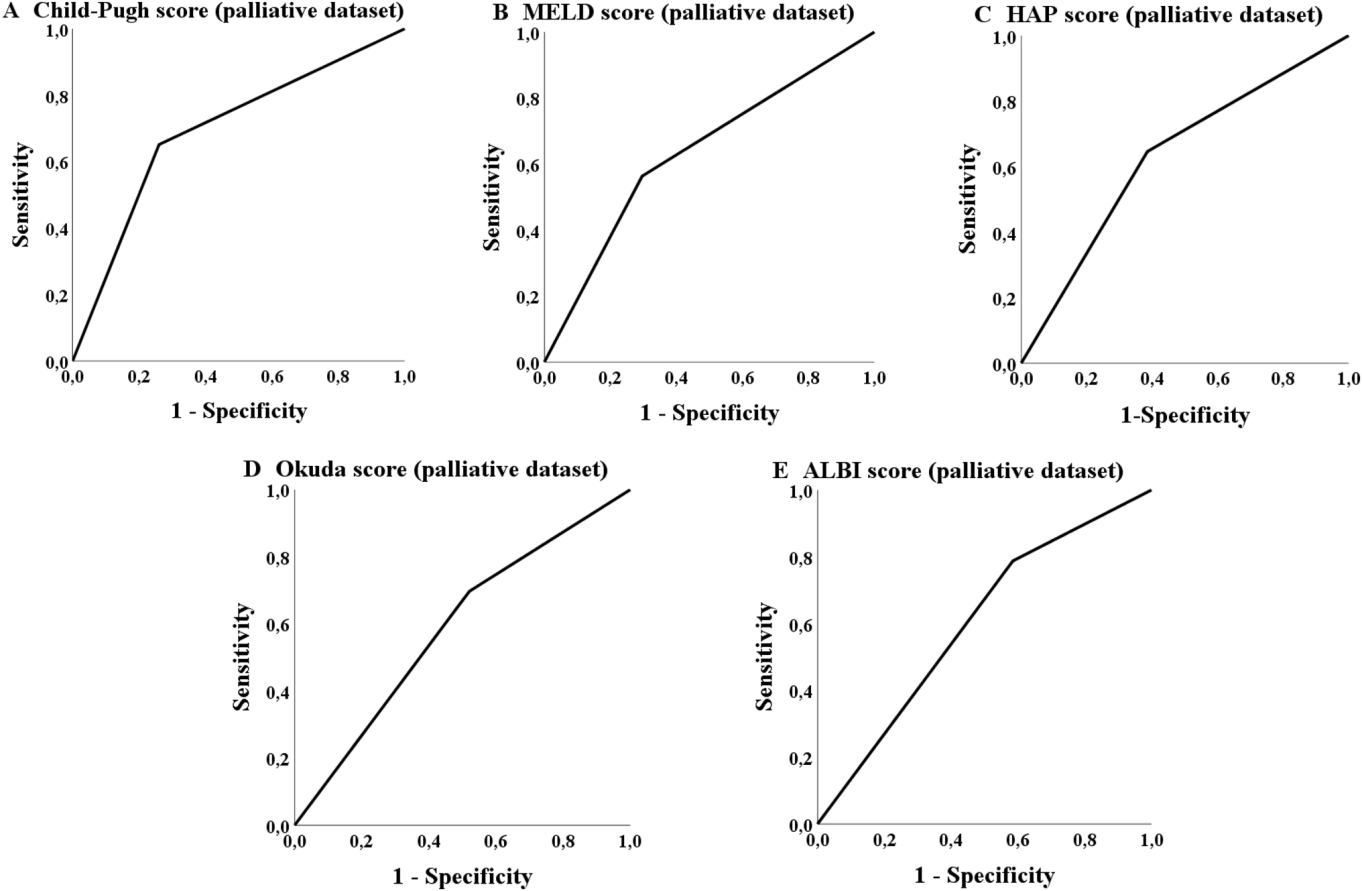
ROC analysis: treatment discontinuation

The ROC analysis in the palliative group showed that five scores achieved a statistically significant *p* value concerning the probability of treatment discontinuation due to adverse events or death (Table 6). The Child–Pugh, MELD-, Okuda-, HAP- and ALBI-score achieved a significant *p* value the AUC values, but did not reach 70% (Table 6; Fig. 4). The most applicable score to predict the probability of a later TACE discontinuation due to the mentioned circumstances was the Child score (class A versus classes B/C). The number of successfully performed TACE procedures differs significantly (*p* = 0.001; Table 7) in overall survival for the palliative cohort (but not in the bridging collective; *p* = 0.354).

#### 3-years survival

**Fig. 5:**
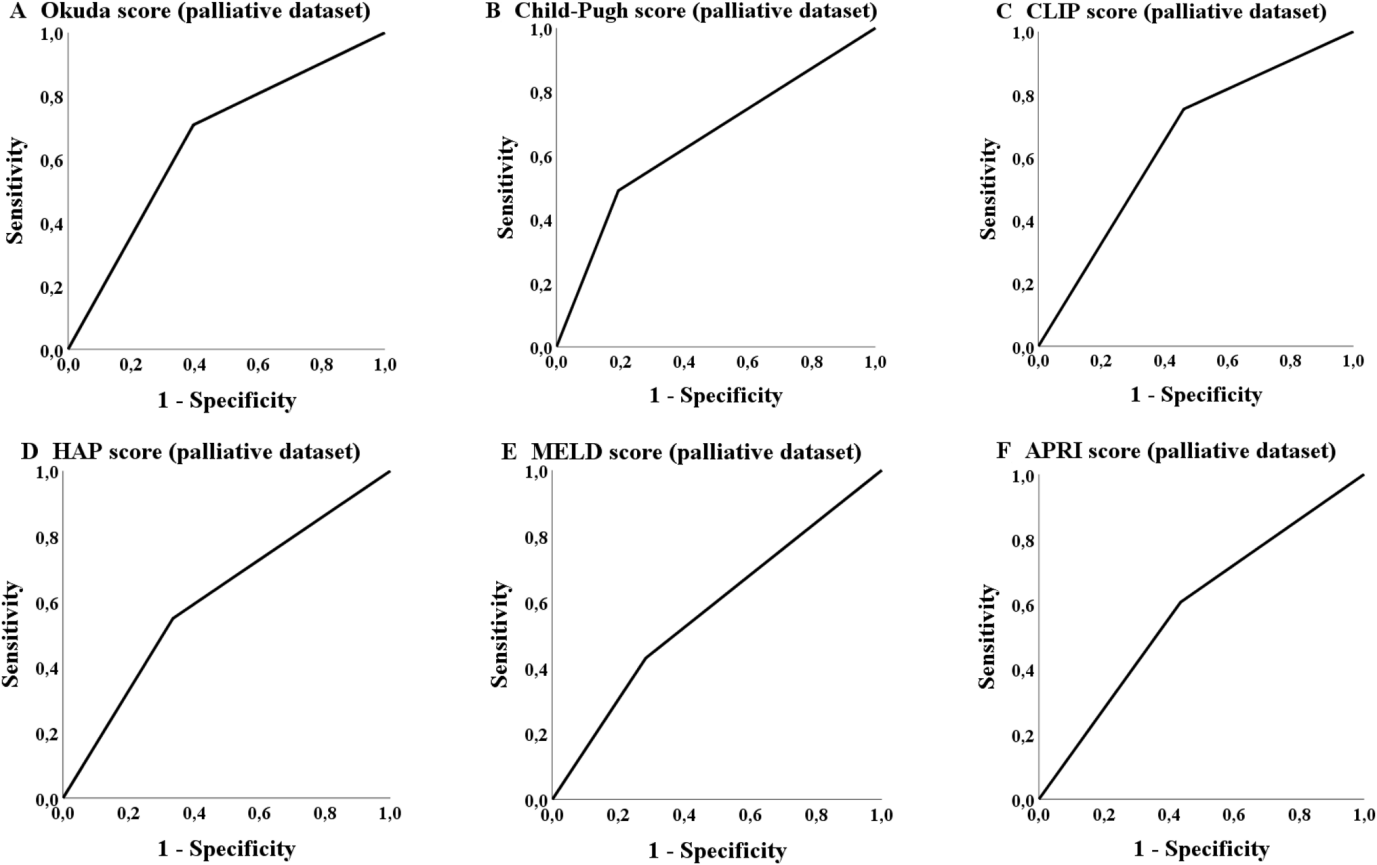
ROC analysis: 3-years survival

The ROC analysis of the database in our study showed that none of the 13 scores had an AUC of over 70% although some of the scores reached significance in the analysis concerning the probability of achieving a 3-years survival such, e.g., CLIP-, Okuda-, HAP- and Child–Pugh score. The APRI score and MELD score also showed statistically significance in the ROC analysis but none of these scores reached an AUC of 60% (Table 5; Fig. 5). The most applicable score to predict the probability of achieving 3-years survival was the Okuda score (stage A versus stages B + C). As in the palliative collective a significant number of patients received TACE in advanced disease stages (BCLC C), an ROC analysis was additionally performed exclusively for BCLC B stage (*n* = 182) but with comparable results (data not shown).

**Table 5:** ROC Analysis concerning 3-years survival

Score	Bridging dataset AUC (sensitivity/1 − specificity)	*p*	Palliative dataset AUC (sensitivity/1 − specificity)	*p*
CLIP group (0–1 vs. 2–4)	0.600 (0.567/0.367)	0.095	0.646 (0.754/0.462)	< 0.001
mART score (< 2.5 vs. ≥ 2.5)	0.542 (0.467/0.382)	0.616	0.520 (0.364/0.323)	0.678
ART score (< 2.5 vs. ≥ 2.5)	0.588 (0.566/0.379)	0.430	0.423 (0.304/0.458)	0.366
ALBI group (A1 vs. A2 + A3)	0.526 (0.800/0.748)	0.661	0.560 (0.689/0.568)	0.080
APRI score (≤ 1.15 vs. > 1.15)	0.577 (0.727/0.574)	0.180	0.585 (0.606/0.436)	0.011
mSNACOR stage (low- and interm. risk vs. high risk)	–	–	0.564 (0.232/0.104)	0.122
SNACOR stage (low- and interm. risk vs. high risk)	–	–	0.503 (0.160/0.154)	0.970
mSNACOR stage (low risk vs. interm risk)	0.484 (0.737/0.768)	0.835	–	–
SNACOR stage (low risk vs. interm risk)	0.455 (0.667/0.758)	0.679	–	–
HAP stage (A + B vs. C + D)	0.552 (0.600/0.495)	0.381	0.607 (0.549/0.336)	0.002
State score (≥ 18 vs. < 18)	0.476 (0.033/0.081)	0.689	0.553 (0.340/0.235)	0.128
Child–Pugh class (A vs. B + C)	0.557 (0.600/0.486)	0.341	0.648 (0.490/0.194)	< 0.001
BCLC stage (A + B vs. C + D)	0.470 (0.118/0.177)	0.594	0.559 (0.357/0.239)	0.063
Okuda stage (A vs. B + C)	0.541 (0.533/0.487)	0.487	0.657 (0.709/0.396)	< 0.001
MELD score (< 10 vs. ≥ 10)	0.544 (0.576/0.487)	0.438	0.573 (0.429/0.284)	0.030

*AUC* area under the receiver operating characteristic curve

### Performance of scores in the bridging dataset

#### Median overall survival (OS)

Only the BCLC score showed significance with regard to median OS in the bridging group (*p* = 0.002) but without decreasing survival time from stage A to stage D (Table 4; Fig. 3).

#### Treatment discontinuation

The bridging dataset does not include TACE sessions of patients who had to stop the general TACE therapy because of adverse events or death (Table 2). Therefore the ROC analysis concerning treatment discontinuation was only calculated in the palliative dataset (Table 6).

**Table 6:** ROC analysis concerning therapy discontinuation

Score	Palliative dataset AUC (sensitivity/1 − specificity)	*p*
CLIP group (0–1 vs. 2–4)	0.565 (0.712/0.583)	0.113
mART score (< 2.5 vs. ≥ 2.5)	0.596 (0.486/0.295)	0.084
ART score (< 2.5 vs. ≥ 2.5)	0.571 (0.500/0.359)	0.533
ALBI group (A1 vs. A2 + A3)	0.601 (0.788/0.585)	0.013
APRI score (≤ 1.15 vs. > 1.15)	0.549 (0.600/0.502)	0.217
mSNACOR stage (low- and interm. risk vs. high risk)	0.581 (0.292/0.129)	0.091
SNACOR stage (low- and interm. risk vs. high risk)	0.632 (0.364/0.100)	0.184
HAP stage (A + B vs. C + D)	0.630 (0.646/0.385)	0.002
STATE score (≥ 18 vs. < 18)	0.548 (0.364/0.269)	0.242
Child–Pugh class (A vs. B + C)	0.696 (0.652/0.260)	< 0.001
BCLC stage (A + B vs. C + D)	0.536 (0.356/0.284)	0.343
Okuda stage (A vs. B + C)	0.588 (0.697/0.521)	0.030
MELD score (< 10 vs. ≥ 10)	0.633 (0.563/0.296)	0.001

*AUC* area under the receiver operating characteristic curve

Receiving an unsuccessful TACE (per patient) does not have influence in overall survival for the bridging (*p* = 0.803) but for the palliative cohort (*p* = 0.046).

#### 3-years survival

In the bridging group the CLIP score reached the best AUC with a value of 60.0%, but there was no significance in the ROC analysis. Furthermore, none of the other scores reached statistical significance concerning the probability of achieving 3-years survival.

## Discussion

Treatment decisions in hepatocellular carcinoma are often complex. In the context of stage migration the assessment of prognostic factors in patients with HCC is crucial for clinical management. For TACE, prognostic scores should provide adequate therapeutic guidance and avoid over-treatment or inadequate response. The aim of this study was a comparative evaluation of the reported panel of scores predicting prognosis of patients undergoing TACE.

Besides the common application of these scores in palliative treatment, the study also evaluated the applicability of these scores for patients undergoing TACE as bridging to LT. Statistical analysis showed that the different scores are not equally applicable in both datasets:

In the palliative dataset most of the scores reached statistical significance for predicting OS, whereas in the bridging dataset, only the BCLC score showed significance. In contrast to the study of Hannover Medical School, in our analysis there was no equally applicable score for both datasets of median OS. However, a significant discriminator regarding prediction of OS between both groups was the number of successfully performed TACE procedures (*p* = 0.001; Table 7) for the palliative cohort (but not in the bridging collective; *p* = 0.354). This is in line with the substantial number of patients suffering from advanced liver disease and enlarged tumor size in the palliative subgroup.

**Table 7:** Kaplan–Meier-analysis: number of successful TACE procedures

Applicability of further parameters concerning the prediction of OS (Kaplan–Meier analysis)	Bridging dataset *n* = 61 Median OS (months) (SD; 95% CI)	*p* (u)	Palliative dataset *n* = 87 Median OS (months) (SD; 95% CI)	*p* (u)
< 2	Not reached	0.354	6.0 (8.4; 0.0–22.4)	< 0.001
2–4	62.0		16.0 (1.1; 13.8–18.2)	
> 4	51.1 (16.4; 19.0–83.2)		41.7 (13.7; 14.8–68.6)	

There is a certain selection bias due to the calculation of different endpoints per TACE rather than per patient. Nevertheless our results of median overall in both datasets (independently of the subgroups of the different scores) are consistent with previous studies (Abbasi et al. 2017; Biolato et al. 2014; Groupe d'Etude et de Traitement du Carcinome Hepatocellulaire 1995; Llovet and Bruix 2003, 2008). In the palliative dataset most scores predict significant differences in median OS. Contrary to current recommendations (Hucke et al. 2014b; Sieghart et al. 2013; Yin et al. 2016), we cannot validate the prognostic power of the ART score neither concerning the endpoint OS nor other endpoints (3-years survival, therapy discontinuation).Various studies also showed that the ART score is not suitable to reflect the OS of patients undergoing TACE in palliative intention (Terzi et al. 2014; Tseng et al. 2015).

The SNACOR score also did not show any applicability concerning all endpoints in our analysis. It was developed in 2016 (Kim et al. 2016) and evaluated in one more study in 2018 (Mahringer-Kunz et al. 2018), in which it also failed to distinguish prognostic subgroups (Mahringer-Kunz et al. 2018). Even though there was no significance of the original score, a certain applicability of the modified version of the mentioned score concerning the endpoint median OS in the palliative dataset was shown. Apart from the ART, mART and SNACOR score, all other scores in the palliative subgroup revealed significant differences of median OS depending on their prognostic groups. These scores may stratify the prognosis of patients undergoing TACE as palliative therapy.

As a result of multivariate analysis only three scores could predict independently median OS of patients undergoing TACE in palliative intention: the mSNACOR, STATE and Child–Pugh score. The applicability of the Child–Pugh score for patients undergoing TACE therapy has been validated in several studies (Brown et al. 2004; Dhanasekaran et al. 2010; El Khaddari et al. 2002; Mondazzi et al. 1994), even though there are also studies indicating that Child–Pugh scoring system is highly subjective (Cholongitas et al. 2005; Durand and Valla 2008). According to our analysis we support the application of the Child–Pugh score for predicting the OS in patients undergoing palliative TACE. The most applicable score to predict the probability of a later TACE discontinuation was the Child score. This appears suitable to the fact of the several laboratory and clinical markers which count into Child score: albumin, INR, bilirubin, encephalopathy and ascites. Although the two last ones are highly subjective, the Child score seems to reflect liver synthesis in case of TACE therapy in palliative intention at its best. Severe impact of liver synthesis is one of the most important reasons of treatment discontinuation besides vascular infiltration.

Due to the missing significances of the ART score in our analysis, we do not support the recommendation of sequential using the STATE score and the ART score to assess the prognosis of patients undergoing TACE (Hucke et al. 2014a). We can support the application of the STATE score at each TACE session for the assessment of OS in patients undergoing palliative TACE treatments. The mSNACOR is also an independent predictor of OS in palliative setting. In general it should be calculated in comparison to the previous TACE instead to the first TACE. Furthermore, it should be calculated at each TACE procedure instead of only at the second TACE. The SNACOR score needs further evaluation (Mahringer-Kunz et al. 2018), due to the fact, that the SNACOR score, in contrast to the mSNACOR score, did not reach any statistical significance concerning the endpoint OS in the palliative dataset. The analysis showed a certain applicability of the Child–Pugh, Okuda, HAP and CLIP score for the assessment of the probability of achieving a 3-years survival after TACE procedure. Nevertheless, none of the scores reached an AUC of more than 70%, which is why a further evaluation or modification of the scores is needed concerning the mentioned endpoint to support clinical decision making. All the mentioned four scores were validated in various studies, but mainly regarding to the endpoint of median OS (Allgaier et al. 1998; Dhanasekaran et al. 2010; Farinati et al. 2000; Georgiades et al. 2006; Kadalayil et al. 2013; op den Winkel et al. 2012; Pinato et al. 2016; Rabe et al. 2003). We recommend that scores should be evaluated concerning further endpoints additional to the endpoint of OS. The probability of discontinuation of TACE therapy due to AE or death is another important endpoint to decide which scores have a prognostic importance. The Child–Pugh score as well as the MELD score showed the best applicability concerning AE or death in our analysis.

The MELD score is an established score especially in patients awaiting LT (Bruns et al. 2014; Kamath et al. 2001), but it may also be useful for predicting certain AE or mortality in patients undergoing TACE procedures (Hinrichs et al. 2017; Sawhney et al. 2011; Testa et al. 2003). According to our analysis, further studies that examine the relation of MELD score before TACE procedure and the probability of discontinuation of TACE therapy would be desirable.

In the univariate analysis of the bridging dataset only the BCLC score was a statistically significant predictor of overall survival, but in contrast to the original publication of the BCLC score (Llovet et al. 1999a), there is no decreasing survival time from stage A (early stage) to stage D (terminal stage), which is shown impressively in Fig. 3. Child–Pugh class C is always accompanied by a BCLC stage D as well as a performance status (PST) stage 1 or 2 is always accompanied by a BCLC stage C. Assuming that a patient has a Child–Pugh class B with, e.g., 9 points at the first TACE, he can be upgraded to 10 points at the second TACE due to a single parameter change. Thus, the patient changes the Child–Pugh class from B to C and is therefore also associated with the BCLC stage D (Llovet et al. 1999a). Accordingly, a patient may also change from a BCLC stage A to a stage D, because a Child–Pugh class A or B does not limit the BCLC score to a specific stage, whereas a Child–Pugh class C is always associated with a BCLC D (Llovet et al. 1999a). However, the Child–Pugh score also includes subjective parameters (Cholongitas et al. 2005; Durand and Valla 2008), why this definition (Child–Pugh C = BCLC D) should be critically scrutinized for patients receiving a TACE as bridging to LT therapy.

The BCLC score has been validated in several studies (Cillo et al. 2004; Llovet et al. 1999a; Marrero et al. 2005; Vitale et al. 2009; Zhao et al. 2015). The *p* value in our univariate analysis of our palliative dataset also suggests that the BCLC score is suitable for assessing survival of patients with TACE treatment. We do not agree with various studies that suggest that the BCLC score is generally suitable for assessing the overall survival of all patients, without making any declaration about therapy indication (Dhanasekaran et al. 2010; Zhang et al. 2014). We do not support the statement that a BCLC stage D is associated with the worst prognosis concerning OS among our analysis, regardless of whether the TACE is performed as bridging to LT or in a palliative intention.

ROC Analysis in the palliative collective reveals similar results for patients with BCLC B in comparison to all other BCLC scores with endpoint treatment discontinuation or 3 years OS (data not shown). Therefore, we conclude that the scores are independent in performance concerning BCLC stadium. Scores do not perform better, if only BLCLC stage B patients are analyzed.

But substantial differences in the performance of the various scores were evident when comparing AUROC in dependence of etiology of liver disease. For the three most frequent etiologies in our cohort (viral, alcoholic and cryptogenic/NASH in descending order) ROC analysis for the endpoints “3 years survival” and “treatment discontinuation” were remarkable different, revealing etiology as a potential confounding factor (Tables 8, 9). Overall performance of the Scoring systems seems to be best for viral etiologies, but poor in alcoholic liver disease patients.

**Table 8:** ROC analysis: treatment discontinuation comparing different etiologies

ROC-analysis concerning 3-years survival	Palliative dataset Viral subgroup *N* = 120 (35.9%)		Palliative dataset Alcoholic subgroup *N* = 96 (28.7%)		Palliative dataset Cryptogenic/NASH subgroup *N* = 59 (17.7%)	
	AUC	*p*	AUC	*p*	AUC	*p*
CLIP group (0–1 vs. 2–4)	0.729	< 0.001	0.504	0.953	0.576	0.382
mART score (< 2.5 vs. ≥ 2.5)	0.589	0.303	0.591	0.302	0.417	0.497
ART score (< 2.5 vs. ≥ 2.5)	0.500	1.000	0.500	1.000	0.643	0.663
ALBI group (A1 vs. A2 + A3)	0.617	0.043	0.463	0.549	0.654	0.064
APRI score (≤ 1.15 vs. > 1.15)	0.584	0.136	0.552	0.400	0.542	0.610
mSNACOR stage (low- and interm. risk vs. high risk)	0.661	0.021	0.545	0.547	0.569	0.759
SNACOR stage (low- and interm. risk vs. high risk)	0.479	0.877	0.543	0.770	0.417	0.739
HAP stage (A + B vs. C + D)	0.707	< 0.001	0.503	0.968	0.693	0.027
STATE score (≥ 18 vs. < 18)	0.564	0.271	0.656	0.012	0.481	0.821
Child–Pugh class (A vs. B + C)	0.712	< 0.001	0.602	0.098	0.674	0.034
Okuda stage (A vs. B + C)	0.766	< 0.001	0.552	0.396	0.551	0.533
MELD score (< 10 vs. ≥ 10)	0.693	0.001	0.507	0.915	0.524	0.769
BCLC score (A + B vs. C + D	0.614	0.032	0.591	0.126	0.395	0.169

**Table 9:** ROC analysis: 3-years survival comparing different etiologies

ROC-analysis concerning therapy discontinuation	Palliative dataset Viral subgroup *N* = 120 (35.9%)		Palliative dataset Alcoholic subgroup *N* = 96 (28.7%)		Palliative dataset Cryptogenic/NASH subgroup *N* = 59 (17.7%)	
	AUC	*p*	AUC	*p*	AUC	*p*
CLIP group (0–1 vs. 2–4)	0.710	0.002	0.503	0.968	0.750	0.037
mART score (< 2.5 vs. ≥ 2.5)	0.600	0.251	0.624	0.240	0.685	0.251
ART score (< 2.5 vs. ≥ 2.5)	0.546	0.767	0.596	0.671	0.643	0.663
ALBI group (A1 vs. A2 + A3)	0.655	0.019	0.550	0.474	0.762	0.028
APRI score (≤ 1.15 vs. > 1.15)	0.601	0.093	0.533	0.635	0.615	0.331
mSNACOR stage (low- and interm. risk vs. high risk)	0.688	0.016	0.456	0.594	0.625	0.424
SNACOR stage (low- and interm. risk vs. high risk)	0.567	0.663	0.617	0.461	1.000	0.127
HAP stage (A + B vs. C + D)	0.691	0.005	0.504	0.953	0.895	0.001
STATE score (≥ 18 vs. < 18)	0.587	0.187	0.486	0.842	0.709	0.077
Child–Pugh class (A vs. B + C)	0.746	< 0.001	0.643	0.039	0.838	0.004
Okuda stage (A vs. B + C)	0.795	< 0.001	0.453	0.500	0.690	0.109
MELD score (< 10 vs. ≥ 10)	0.670	0.008	0.517	0.805	0.817	0.007
BCLC score (A + B vs. C + D	0.552	0.398	0.504	0.953	0.532	0.787

In general, the ROC analysis for both groups revealed that there is no score reflecting a sufficiently selectivity to make clear clinical decisions. This is probably influenced by the fact that a TACE procedure is still not sufficiently standardized. Neither concerning the type of intervention (conventional, DEB, biodegradable), nor the frequency of the TACE procedures or regarding to the different subsequent therapies (RFA, Sorafenib, BSC etc.) are currently standardized selection criteria. According to the results of the bridging dataset further evaluations and modifications of scores are needed, especially for patients receiving TACE procedures as bridging to LT therapy.

## Conclusion

The characteristics as well as the outcome of patients receiving TACE are significantly different depending on the therapy indication. In contrast to previous evaluations, scoring for OS after TACE should be separately evaluated for curative (LT) and palliative settings. Regarding TACE as palliative therapy the Child–Pugh score, STATE score and mSNACOR score performed best for the prediction of median OS. In contrast to other studies we could not validate a prognostic power of the ART score. Furthermore, the SNACOR score was only informative, when directly comparing serial, respectively, when it is calculated such as the mSNACOR.

Overall, none of the evaluated scores seems to be promising in terms of clinical decisions making with respect to stage migration in both cohorts. Only the BCLC score was able to predict the OS probability in the bridging dataset but without decreasing survival time from stage A to stage D. We conclude that further efforts are needed, especially in patients undergoing TACE as bridging to LT, to establish appropriate criteria for making valid predictions and thus support decision making processes in daily clinical routine.

## Abbreviations

TACE: Transarterial chemoembolization
HCC: Hepatocellular carcinoma
LT: Liver transplantation
CLIP: Cancer of the Liver Italian Program
ART: Assessment for retreatment with transarterial chemoembolization
ALBI: Albumin-bIlirubin grade for HCC
APRI: AST to platelet ratio index
SNACOR: Tumor size and number, baseline alpha-fetoprotein, child-pugh and objective radiological response
HAP: Hepatoma arterial-embolisation prognostic score
STATE: Selection for transarterial chemoembolization treatment
MELD: Model of end stage liver disease
BCLC: Barcelona clinic liver cancer staging system
